# The *Rosetta Stone* of interactions of mucosa and associated bacteria in the gastrointestinal tract

**DOI:** 10.1097/MOG.0000000000000992

**Published:** 2023-11-20

**Authors:** Serena Berberolli, Mengqi Wu, Francisco M. Goycoolea

**Affiliations:** School of Food Science and Nutrition, University of Leeds. Leeds, LS6 4RG, United Kingdom

**Keywords:** gut microbiota, mucosa, O-glycans

## Abstract

**Purpose of review:**

Gut microbiota–mucosa–epithelial cells co-exist in an intricate three-way relationship that underpins gut homeostasis, and ultimately influences health and disease conditions. The O-glycans of mucin glycoproteins have been uncovered as a centrepiece of this system, although understanding the phenomena at play at the molecular level has been challenging and subject to significant traction over the last years. The purpose of this review is to discuss the recent advances in the phenomena that mediate microbiota and mucus multidirectional interactions in the human gut.

**Recent findings:**

The mucus biosynthesis and degradation by both commensal and pathogenic bacteria is under tight regulation and involves hundreds of carbohydrate-active enzymes (CAZy) and transporters. The fucosylation of O-glycans from mucin-2 seems to dictate binding by pathogenic species and to influence their virulence. Less clear is the influence of O-glycans in quorum sensing and biofilm formation. We have reviewed the advances in the *in vitro* models available to recreate the phenomena that capture the physiological context of the intestinal environment, emphasising models that include mucus and other aspects relevant to the physiological context.

**Summary:**

The recent findings highlight the importance of merging advances in analytical (glycans analysis) and omics techniques along with original robust *in vitro* models that enable to deconstruct part of the high complexity of the living gut and expand our understanding of the microbes-mucosa relationships and their significance in health and disease.

## INTRODUCTION

Mucosa is an essential component of the human gut microbiome that shields the epithelial cells’ glycocalyx [1^▪▪^]. It acts as a selectively permeable physical barrier and an ecological niche that provides nutrients and compartmentalises the host's microbes and the immune response. It plays a crucial role in spatial differentiation to maintain microbial diversity and coexistence across and along the gut. Mucin-2 (MUC2), the main constituent of the gut's mucosa, is a large glycoprotein rich in O-glycans covalently bound to the protein core. O-glycans make up to 80% of the weight of MUC2 and collectively give rise to hundreds of structurally unique O-glycans built that extend out to interact with pathogens. All these properties and the integrity of mucus are highly relevant to gut homeostasis and health status [2^▪^]. Healthy mucosa is a dynamic barrier under a continuous process of biosynthesis, secretion, posttranslational modification and clearance. The mucosa is known to harbour a mucus-associated microbial community (MAMC) comprised of specific bacterial families that evolved to adapt to specific posttranslational modifications in the gut mucosa, and its diverse glycotypes encode for many carbohydrate-active enzymes (CAZy) [1^▪▪^,^3^,^4^,^5^^▪▪^]. The nature of the relationship between microbes and the mucosal environment spans the spectrum from symbiotic (mutualistic), commensal, and pathogenic (parasitic). The repertoire of glycans expressed on the host cell surfaces, the mucosal interface and the microbes are known to influence every aspect of the type of such interactions at play [6]. In this opinion, we seek to provide a critical updated overview of the underpinnings phenomena that mediate microbiota and mucus interactions in the human colon, namely the regulation of mucus biosynthesis, the influence of pathogenic bacteria on mucosal integrity and conversely how mucin O-glycans influence pathogenic bacterial virulence. We have also reviewed the advances in the *in vitro* models available to recreate the phenomena that capture the physiological context of the intestinal environment, with attention on models that include mucus and other aspects relevant to the physiological context. 

**Box 1:**
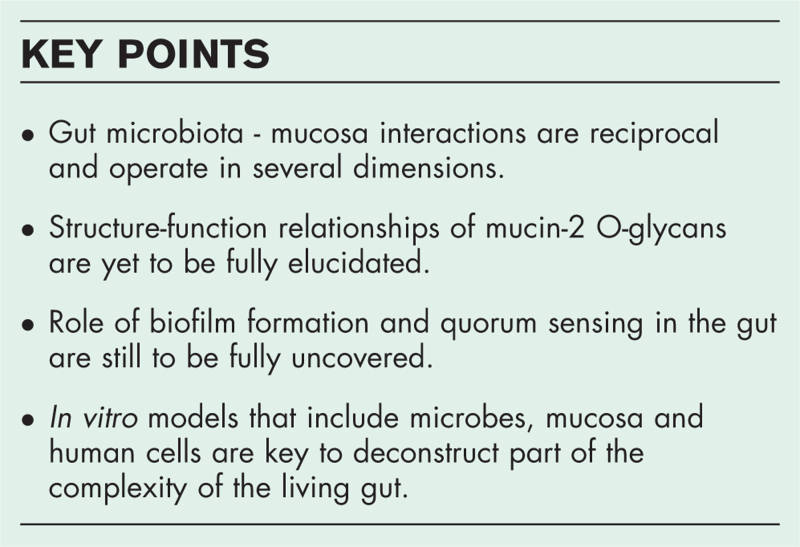
no caption available

## DECIPHERING THREE-WAY INTERACTIONS BETWEEN MICROBES–MUCOSA–EPITHELIUM

**FIGURE 1:**
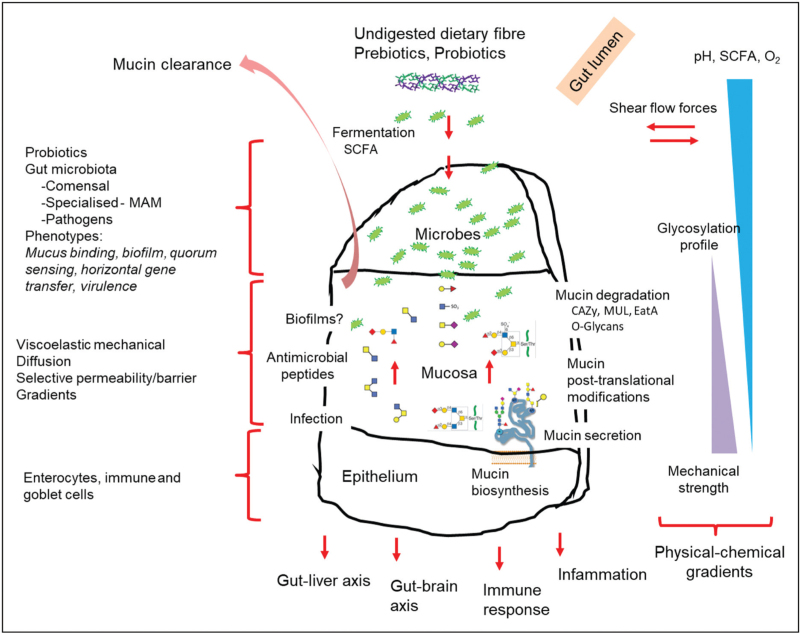
Schematic simplified representation of the multidirectional spatiotemporal relationships between the gut microbiota-mucus microenvironment-epithelium. The complexity of the phenomena is analogous to deciphering a *Rosetta Stone* sitting at the centrepiece of this analogy. The ‘hieroglyph cartouches’ can be paralleled with the cues encripted in the structure of the O-glycans of the mucin glycoproteins, the bacterial carbohydrate-active enzymes (CAZy), the mucin-utilisation loci (MUL) and autotransporters (EatA) needed to decode them. Other catabolic products and metabolites diffusing from the lumen into the mucosa include the fermentation products of dietary fibre (dietary glycans, SCFA).

Mucus–bacteria relationships are also known to have a direct influence in bacterial phenotypes that entail among others, the capacity to bind onto (e.g., *Lactobacillus fermentum* and *Bacteroides thetaiotaomicron*), diffuse through (e.g., *Helicobacter pylori*), degrade (e.g., *Akkermansia muciniphila*), obtain fucose (e.g., *B. thetaiotaomicron*) and form biofilms within (e.g., *Pseudomonas aeruginosa*) the mucosal microenvironment. Understanding the intricate physical (e.g., mechanical strength, shear and peristaltic flow forces, diffusion, viscoelasticity), chemical (e.g., O_2_, pH, metabolites – SCFA, bile salts, antimicrobials) and biochemical (e.g., O-glycans) stimuli and gradients that exist across the stratified and loose mucosal layers, poses a considerable challenge, and the underpinning mechanistic aspects at play have remained elusive. The three-way directional interactions between the gut microbiota-mucus microenvironment and epithelium can be compared with deciphering a *Rosetta Stone*. Under this analogy, the O-glycans structure encoded in the heavily glycosylated mucin proteins are paralleled to the ‘Egyptian hieroglyph cartouches’ (Fig. 1).

## MUCUS BIOSYNTHESIS/REGULATION AFFECTED BY BACTERIA

As a protective layer, dysregulation of mucus integrity could lead to pathogens invasion and infection. Hence, preserving its integrity is essential for gut homeostasis and health [1^▪▪^]. Besides CAZy and the mucin utilisation locus (MUL) involved in the degradation and catabolism of mucins (1,5), carbohydrate autotransporters are involved in mucin degradation. A recent study identified EatA, an autotransporter protein which relates to the degrading of MUC2, thus leading to the accessibility of mucin to pathogenic species enterotoxigenic *Escherichia coli* (ETEC) [7] – how mucins are relevant to health and disease will be discussed further in a later section.

## BACTERIA AND MUCIN GLYCAN UTILISATION

Several recent reviews have addressed the biosynthesis and regulation of mucin [1^▪▪^,8^▪^,^9^,^10^,11^▪^]. Given that mucins are comprised of highly O-glycosylated proteins of four main types (Core 1, Core 2, Core 3 and Core 4), the interaction between mucin O-glycans with gut bacteria is the cornerstone of the ongoing efforts to elucidate the enzymatic degradation, signalling and other roles of these glycans. Here, we first focus on how commensal bacteria utilise mucin glycans. Inspection of the CAZy database (http://www.cazy.org/), shows that the enzymes involved in mucin degradation belong to the family of glycoside hydrolase (GH) family, either from sialidases or fucosidases type. These enzymes can break down mucin and release monosaccharides available to the commensal bacteria and other nonmucin degraders. This study also reveals that commensal species such as *A. muciniphila*, *Bifidobacterium bifidum*, and *B. thetaiotaomicron* have more mucin-associated GH compared to other commensal bacteria that have weaker mucin-degrading ability [11^▪^]. *A. muciniphila* is the most promising species that has been the focus of recent research. *A. muciniphila* has shown the capacity to degrade mucin via constitutive expression of MUL genes. These genes have been hypothesised to encode many proteins that single out *A. muciniphila* from other mucin degraders [12]. Another study on *A. muciniphila* has uncovered the mucin degrading mechanisms were associated with two enzymes, the AmGH29C and AmGH95B, which contribute to less α1,3/4- and α1,2-fucosylation [13^▪▪^]. The use of next-generation gene sequencing transcriptomic techniques to uncover the key genes associated with mucin utilisation in the commensal species has been instrumental. Further studies geared to discover new species relevant to health and specific food-related health conditions and noncommunicable diseases, using *in vitro* and *in vivo* approaches, as well as the use of machine learning and big data analytics, are expected to expand the current knowledge.

## MUCIN BIOSYNTHESIS ASSOCIATED WITH PROBIOTICS

The biosynthesis of mucin is vital for a healthy gut, and it has been associated with different factors. We highlight here the recent studies that have addressed the presence of probiotics into the mucus environment. The metabolites of *Lactobacillus rhamnosus* GG culture have been suggested to upregulate the production of MUC2 in mouse colon [14^▪^]. This pinpoints the potentials of probiotics administration for health benefits. Another study explored the roles of two other probiotics, *Lactiplantibacillus plantarum* Q7 and *L. plantarum* F3-2, in MUC2 production. These two strains were found to promote the MUC2 production by upregulating the genes that encode tight junction proteins in mouse models [15]. These results suggest the potentials of the probiotics in promoting human health. Mucin degrader *A. muciniphila* could also serve as a probiotic for health conditions such as obesity. However, research in mouse model that applied with excessive amount of *A. muciniphila* showed a thinner intestinal mucus layers, as well as fewer tight junction protein production [16^▪^]. This study highlights the importance of being cautious about applying these bacteria species as therapeutic agents.

## MUCUS INTEGRITY SHAPES GUT MICROBIOTA COMPOSITION

### Pathogen binding

The mucosa acts as a first line of defence against infection by pathogenic species. Of note, pathogens associated with mucosa share the capacity to bind the mucus and subsequently colonise the host. As the main functional component of the mucosal layer, mucins are related to interactions with various bacterial species.

*H. pylori* is a pathogen that colonises nearly half of the world's population, and in some regions its prevalence is up to 80%. It triggers chronic gastritis and stomach ulcers in a small subset of individuals. The patterns of glycans expression in both the host and the microbe seem to determine whether *H. pylori* remains as a commensal or triggers disease pathology. Expression of adhesine BabA is known to determine the capacity to bind the gastric epithelium expressing glycans that terminate with the Lewis-b blood group antigen. This, however, is limited to mucus-producing pit cells [6]. This result has been confirmed in mucin samples from either *H. pylori-*infected or noninfected individuals using LC-MS and MS/MS. A higher binding resulted in the Leb-positive mucin samples from *H. pylori-*infected stomach [17^▪^]. The same study also found that fucosylation of mucins appeared to be more relevant in the *H. pylori-*infected samples. *H. pylori*, similar to *B. thetaiotaomicron*, has evolved mechanisms to obtain fucose from its host and stimulates the expression and secretion of α-l-fucosidase 2 (FUCA2) [6]. Yet another study used a microarray library of selected fucosylated mucin glycopeptides to determine the specific binding motifs for *P. aeruginosa* lectin LecB and *Clostridium difficile* toxin A [18^▪^]. These studies concur to suggest that the fucosylated glycans are relevant for pathogens binding and should be the focus of further attention.

## BIOFILM FORMATION

Biofilm formation is a mechanism that promotes single bacterial and bacterial communities attachment to host surfaces that provides them with resistance to host immune clearance and antibiotics treatment. Colonisation and biofilm formations by pathogenic species in the mucus vicinity could lead to later inflammatory diseases. However, research in this area has been scarce. A study has documented the use of live imaging to record the biofilm formation of *P. aeruginosa* using a tissue-engineered airways model. It was found that mucin surface spatial distribution contributes to the early induction time of the cell-to-cell communication and biofilm formation mediated by soluble signalling quinolone signals (known as *quorum sensing* (QS)) among the nonmucoid *P. aeruginosa* strain [19^▪^]. This study seems to contradict the one by Wheeler *et al.* reporting that mucus degradation glycans abrogate virulent responses of *P. aeruginosa*[20], in agreement with similar findings in *Vibrio cholera*[21]. Only future studies will shed light into the mechanistic aspects that dictate the host-pathogen relationships and the role that mucosal interface plays.

Another *in vivo* study in the intestinal crypts addressed the biofilm formation of *E. coli*. Using a mouse model, they found that α1,2-fucosylation of intestinal mucus can suppress *E. coli* biofilm formation in the crypts [22^▪▪^]. In connection with the previous section on pathogenic bacterial binding, fucose and fucosylation pathways seems to be a promising target for attenuating pathogen virulence. Overall, there are only very few studies that has been focused on the interactions between mucus and bacterial biofilm formations, thus highlighting a gap in research for further exploration.

## REGULATION OF BACTERIAL VIRULENCE BY MUCIN O-GLYCANS

Mucus degradation glycans on microbe-epithelium signalling processes have been found to abrogate virulent responses such as in *P. aeruginosa*[21], *V. cholera*[22^▪▪^], and *Streptococcus mutants*[23^▪^] via inhibition of QS, known to drive the virulent response of pathogenic bacteria including the expression of flagella, exopolysaccharide and biofilms formation. How these collective phenotypes influence colon homeostasis and the delicate balance that governs symbiosis and dysbiosis processes is also poorly understood. Only future research will shed light on whether this is an avenue worth pursuing to deal in a rather exquisite way with the virulence of specific pathogens while overcoming with antimicrobial resistance.

## *IN VITRO* MODELS

Since their original inception in 2016, microfluidic gut-on-chip platforms have emerged as powerful research tools to partially deconstruct the complexity that operates *in vivo* and address the role of phenomena such as varying mechanical forces, fluid flow, and oxygenation conditions while capturing other dynamic aspects of the gut's physiological context by incorporating human cells and microbes [24^▪▪^]. While these approaches have shown to be powerful and robust, they are complex to build and operate and rely on synthetic polymers such as polydimethylsiloxane (PDMS), which is prone to adsorption by the chemical compounds present.

Other recently documented *in vitro* models that have sought to account for the inclusion of mucus propose an electrospun gelatin structure coated with mucin with a view to support the formation of biofilms [25,26^▪^]. It was found that mucin does not change the adhesive ability of gut microbiota, since the gut microbiota can form biofilms on both mucin-coated and mucin-free electrospun gelatin structures and the total amount of adhered microorganisms is maintained over time. However, the mucin coat induced an increase in the abundance of mucus-associated bacteria such as *Akkermansia*, *Lactobacillus* and after 72 h, of *Faecalibacterium*, possibly due to cross-feeding on mucin degradation by the earlier colonisers. This approach highlights the potential of biomimetic biomaterial studies and technologies such as electrospinning to develop scaffolds amenable for *in vitro* studies.

An original multicellular flipwell 3D co-culture system was documented to model the mucosal microenvironment comprising epithelial cells (Caco2 and HT-29/MTX mucus producing cells) and differentiated THP-1 monocytes differentiated into polarised macrophages [26^▪^]. The human cells are grown on sandwiched PET microporous membranes. *Bacillus subtilis*, a probiotic bacterium, was introduced in a separate compartment for a limited time, long enough to prove the concept of the induction of the secretion of mucus. Its ease of construction, low cost and capacity for high throughput analysis are clear assets of this setup.

Another documented *in vitro* model that represents a step forward toward more physiologically representative models, is a vertical diffusion chamber (VDC) [27^▪▪^], able to provide a microaerobic environment that allows to culture most of gut microbiota bacterial species, which are predominately anaerobic. Moreover, the introduction of mucin-producing cells (LS174T) allows to study the mucin binding capacity (e.g., *Limosilactobacillus reuteri*) and the glycan degradation (e.g., *Ruminococcus gnavus*) of commensal species and their effect on preventing pathogens infections, such as enteropathogenic *E. coli* (EPEC).

Using a bioinspired approach, Sardellli *et al.*[28^▪^], developed a 3D dynamic *in vitro* model that mimics mucus flow and turnover. A biomimetic mucus material was developed by crosslinking mucin with alginate. By using *E. coli* as a model gut species, it was possible to study the behaviour of bacteria embedded in the mucus matrix and inoculated on top within a dynamic model, where bacterial growth is maintained without the need of medium refresh.

The recent progress in studies aiming to make *in vitro* models more sophisticated, namely to co-culture bacteria with other elements (mucus, cell, immune cells, etc.) and study the crosstalk, while in anaerobic conditions. Current efforts have been focused on overcoming the limitations of microfluidic lab-on-chip approaches and enable less expensive, dynamic conditions of the gut. *In vitro* models still fail to reproduce important aspects of the physiological context such as peristaltic movements and shear flow forces, hormonal and neural control, the complex anatomy of the gut and the changes in behaviour over time within the human colon lumen.

## CONCLUSION

In this opinion we have discussed the recent progress on the relationships between gut microbiota and mucosa. Despite the significant progress achieved in elucidating the role of O-glycans from mucin and the diet, there are still huge gaps in our understanding of structure-function relationships at play. We are confident that efforts will continue in this regard aiming to decipher the chemical code that mediate and regulate bacterial phenotypes and prevent infection. Overall, there are only very few studies that have been focused on the interactions between mucus and bacterial biofilm formation, and on the role of bacterial cell-to-cell communication via QS in mediating the virulence of pathogenic bacteria. This is yet another gap in research for further exploration. *In vitro* models still fail to reproduce important aspects of the physiological context such as peristaltic and shear flow forces, hormonal and neural control, the complex anatomy of the gut and the changes in behaviour over time within the living human colon lumen. Further studies geared to uncover new species relevant to health and specific food-related health conditions and noncommunicable diseases, using *in vitro* and *in vivo* approaches, as well as the use of machine learning and big data analytics, are expected to contribute greatly to decipher the glycans language of the gut, continuing to expand our current understanding.

## Acknowledgements

*This work was supported by EPSRC Centre for Doctoral Training in Soft Matter for Formulation and Industrial Innovation (SOFI2; EP/S023631/1) in the form of a full PhD scholarship for Serena Berberolli.*

### Financial support and sponsorship

*None.*

### Conflicts of interest

*There are no conflicts of interest.*

## REFERENCES AND RECOMMENDED READING

Papers of particular interest, published within the annual period of review, have been highlighted as:

▪ of special interest
▪▪ of outstanding interest
